# The Hospital. Nursing Section

**Published:** 1905-05-13

**Authors:** 


### The Hospital.

### Nursing Section.

Contributions for this Section of "The Hospital" should be addressed to the Editor, "The Hospital" Nursing Section, 28 & 29 Southampton Street, Strand, London, W.C.

No. 972.—Vol. XXXVIII. SATURDAY, MAY 13, 1905.

### Notes on News from the Nursing World.

### COLONIAL NURSING ASSOCIATION.

The annual meeting of the Colonial Nursing Association will, by permission of the Duke and Duchess of Marlborough, be held at Sunderland House on Wednesday, June 7th, at 3.30 p.m. Princess Henry of Battenberg has signified her intention to be present, and the Earl of Westmeath will preside. The speakers will be the Secretary of State for the Colonies, Mr. Haldane, M.P., and Lady Balfour of Burleigh. As this will be the first occasion of Mr. Alfred Lyttelton's appearance on the platform of the Association, his speech will be looked for with considerable interest. When Mr. Chamberlain was at the Colonial Office he rendered, as our readers well know, personal assistance to the Association, and Mrs. Francis Piggott, its originator and founder, would be the first to acknowledge the debt which it owes to Mr. Lyttelton's distinguished predecessor. We hope that the present Colonial Secretary will be able to infuse fresh vigour into its management, not only by speeches at meetings, but also by manifesting personal interest in its objects.

### THE REGISTRATION BILLS.

Both the Bills for the registration of nurses were among the Orders of the Day on Tuesday; but, of course, neither of them was reached, or had the remotest chance of being discussed on a night which the promoters do not appear to realise belongs to the Government after the Easter recess. We understand that even if an opportunity were found of raising a debate on the question, several members would decline to vote for either of the measures, not so much because of the principle involved, but because they are of opinion that while the Select Committee is sitting an attempt to obtain legislation cannot be justified.

### THE LAMBETH HOME NURSING SCHEME.

With regard to the scheme which the Lambeth Guardians have prepared for the home nursing of sick paupers, we are informed that they have not yet decided upon any details. At the present moment they are awaiting the approval of the Local Government Board to the principle of their proposal for placing the out-door sick poor of certain districts under the immediate supervision of the chief medical officer of Lambeth Infirmary, with one or two assistants, in lieu of out-door district medical officers. The proposal, we understand, has been considerably delayed by the procedure necessary to determine the present appointments.

### THE QUESTION OF A MATRON'S AUTHORITY.

The Board of Management of Swansea Hospital has held a special meeting in order to investigate a complaint made by the matron against the medical officer, whom she accuses of sending a nurse off duty without consulting her; she also alleged that the house surgeon had sent a nurse off duty without informing her at the time or subsequently, and that the medical officer supported the action of the house surgeon. The reply of the house surgeon, Dr. Florence Price, was that she did not know it was the rule to inform the matron in such a case, but the medical officer justified the course he had pursued on the ground that the nurses had been on duty when they were not in a fit condition, and he averred that when he ordered them off duty he told the sister to "let the matron know." After hearing statements from all parties, the Board, by a majority of 14 to 4, adopted a conclusion which had previously been arrived at by the House Committee to the effect, "That it rests with the medical officer to decide whether a nurse is in a proper state of health to be on duty, and that when the medical officer decides that a nurse is not in a fit state for duty the matron should be immediately informed." We think that the medical officer was able to show that nurses were on duty who were obviously unwell, and were therefore unfit to be on duty. One was suffering from two discharging whitlows on her fingers, and another from acute colic. But the matron should have been asked at the time to explain why they were on duty. She is the proper person to call to account for either the conduct or the condition of the nurses under her charge. We are surprised that the matron of Swansea Hospital is not satisfied with a decision which, in our opinion, is the only one possible in the circumstances.

### THE NEW MATRON OF ADDENBROOKE'S HOSPITAL.

A notable appointment to the vacancy at Addenbrooke's Hospital, Cambridge, has been made in the person of Miss Alice Blomfield, the resident lady-superintendent of the East End Mothers' Home, the well-known institution in Stepney. Miss Blomfield was trained at the London Hospital, where she was holiday sister, and after serving as sister at Queen Charlotte's Hospital, she undertook the important post which she now relinquishes in order to become matron of Addenbrooke's. The special feature at the East End Mothers' Home is the training of midwives and monthly nurses, and under the direction of Miss Blomfield the usefulness of the institution has been largely increased.

### POOR-LAW PROGRESS

The Leicester Guardians are advertising for a matron for their fine new infirmary at North Erington, and it is pleasing to note that they offer satisfactory remuneration. The salary the first year is to be £130, rising by £10 per annum to £150, with rations, furnished apartments, washing, and uniform. It is also signified that candidates, who must be fully-trained and certificated nurses, should be experienced in the training of probationers and in the management of domestic arrangements and control of the staff of a hospital or similar institution. The possession of a midwifery certificate is likewise a *sine qua non.* Under these circumstances there is no reason why the Leicester Guardians should not secure the services of a highly capable and experienced woman.

### PROPOSED GOVERNMENT BADGE FOR NURSES.

A registered midwife has been prompted by the correspondence in our columns on the question of uniform to come forward with a novel proposal of her own. She suggests the registration of nurses' uniform, the evidence of registration being the wearing of a badge issued by the Government. According to her plan, this would involve the creation of quite a new department, for she urges that medals of no fewer than seven designs should be struck in order to carry out her idea. Her argument is that if this could be carried out the public would be able "to tell at a glance whether or not the owner of the uniform had the right to wear it." Apart from other reasons which render the scheme impracticable, does the author know so little of the public as to imagine that they would take the trouble to acquaint themselves with the special significance of seven badges?

### QUEEN'S NURSES AND CHURCH COLLECTIONS.

It is unusual for a Nursing Association to depend for its income upon the support of church collections. This, however, is the case at Galashiels, and at the annual meeting of the Nursing Institute in that town one of the speakers said that "he hoped there would be always sufficient from this regular source of income to meet the expenses." The inference is that the whole community at Galashiels are churchgoers. If this be not so, it does not seem fair that people who do not go to church should not be asked to show appreciation of the work of the Queen's nurses. One grateful patient at Galashiels contributed ten shillings; there should be several of these, though we can understand the satisfaction entertained by the Provost, because there is no need in this instance to canvass for subscriptions. We are afraid that, but for persistent canvassing, many associations would find it very difficult to pay their way at all.

### THE IRISH BENEVOLENT FUND FOR NURSES.

The general meeting of King Edward the Seventh Coronation National Fund for Nurses will take place in Dublin on the 24th instant, and among the speakers will be Miss Hampson, lady superintendent of Portobello Hospital, and Miss Kelly, lady superintendent of Stevens' Hospital. The nurse members of the Society are earnestly requested to attend. We observe that this organisation has been doing a little bit of relief. At a special meeting of the Council an application for help by one of the nurse members was discussed, and after careful consideration of the case it was decided to grant her the sum of ten pounds.

### NORFOLK COTTAGE NURSES.

In a letter from Lord Lindley, read at the annual meeting of the Norfolk District and Cottage Nursing Federation, of which he was until lately chairman, it was suggested that the committee should affiliate with the Association for Promoting the Training and Supply of Midwives. The suggestion was favourably received, and meanwhile the committee propose that parish meetings should be held in order to consider the situation in view of the operation of the Midwives Act. It is quite realised that the days of the untrained midwife are over, but there are difficulties in Norfolk, as in other counties, in the way of providing an adequate number of persons likely to be certificated by the Central Midwives Board.

### COMPLIMENTS TO A HOSPITAL STAFF.

The annual meeting of the subscribers of the Norwood Cottage Hospital was held on Saturday, the president, Mr. Tritton, M.P., in the chair. In the course of his speech Mr. Tritton observed that while dealing with the personnel of the hospital he could not refrain from saying how much they owed to the matron and nursing staff for their services during another and exceptionally busy year. He had often wondered at the zeal, energy, and kindness which they threw into the nursing of the sick. Subsequently, Dr. Gandy moved, "that the thanks of this meeting be given to the matron, Miss Clark, and the nursing staff for their continual kindness and attention to the patients," and the resolution was seconded, supported, and carried with applause.

### A NEW CLUB FOR NURSES.

There has just been opened at 129 St. George's Road, S.W., a residential club for nurses. The house is comfortable and homelike; the dining-room and sitting-room are airy, bright rooms, sufficiently large but without the gaunt and swept-out appearance so typical of an "institution." The house, which can accommodate 20, is somewhat old-fashioned, with good-sized rooms which can be divided in order to form cubicles. The divisions are effected by bright curtains, letting in air and light, and the terms for cubicles are 17s. 6d. per week, which includes full board. Private rooms are charged respectively £12 and £24 yearly. The latter are shared between two friends. For private rooms the charge for board is 10s. 6d. Night nurses are accommodated at the top of the house to insure quiet, and the price per week for bed and one meal, namely, 10s. 6d., is within the means of most. Meals on isolated occasions are really cheap. For instance, a penny for a cup of tea, coffee, or cocoa, or threepence with bread and butter. The hours of meals have a certain latitude, the rules are few and simple, and the entrance fee to the club is a guinea. Boots are cleaned for a halfpenny a pair, baths are charged twopence; meals taken in bedrooms threepence extra; boxes can be housed for threepence a week, and scuttles of coals for bedrooms are sixpence each. A member may invite a friend to meals on the same terms as the inmates, and, altogether, the club seems likely to supply a want.

### GIFTS TO THE KENT NURSING INSTITUTION.

An interesting feature in the yearly report of the Kent Nursing Institution, which was read at the annual meeting last week, was the announcement that a piano for the nurses' sitting-room had been presented to the home through Nurse Brunt, a former member of the staff. It had been intended to hold a sale of work in West Malling for this purpose and, when the presentation was made, at the suggestion of the president, fresh notices were sent out announcing that the sale would still be held and that the proceeds would be devoted to obtaining several articles of furniture and other comforts for the nurses. As a sum of £36 10s. was realised by the sale a number of these articles were procured, and the committee were desired by the lady superintendent and nurses to express their hearty thanks to all who assisted. In their report, which showed a very satisfactory result, the committee state that it could not have been attained but for the energetic co-operation of Miss Mottram and her staff.

### QUEEN'S NURSES AT HEREFORD.

At a special meeting of the Herefordshire County Nursing Association, it was decided to affiliate the organisation to the Queen Victoria Jubilee Institute. After Lady Biddulph had recapitulated the proceedings which led up to the decision, Miss Amy Hughes delivered a clear and interesting address. In the course of her remarks, Miss Hughes mentioned that in the case of the counties already affiliated to the Jubilee Institute, the system of joint supervision exercised by the union of the duties of inspector of midwives and county nursing superintendent in one person, was working well. In addition to the fully-trained nurses employed by the Association, who are now, of course, recognised as Queen's Nurses, a number of village nurses also work under its auspices in different districts, and the seventh of these will begin her training next month.

### HOME OF REST AT SCARBOROUGH.

Nurses in search of change and rest may be glad to hear that they will be made very welcome at the Yorkshire Convalescent Home and House of Rest, South Cliff, Scarborough, of which the lady superintendent is a trained nurse. This home was opened 34 years ago for ladies of limited means who are engaged in professional work, for those whose working days are past, and for those who not being obliged to work are yet unable to get a much needed change. In certain periods of the year ladies in much reduced circumstances are given free admission, and in others they are received on very moderate terms. The home is undenominational, and while every care is given to those in need of nursing, actual invalids are not accommodated. This deserving institution is perhaps not sufficiently well known, although we believe that the visitors are drawn from all parts of the Empire.

### IRISH NURSES' ASSOCIATION.

At a meeting of the Irish Nurses' Association last week a lecture was given by Sir Andrew Read on "Glimpses of Egypt," Miss Kelly, lady superintendent of Steevens' Hospital, Dublin, presided, and the large number of nurses present were greatly interested in the lecture, which was illustrated by lantern slides. Miss Manning proposed, and Miss A. Roberts seconded, a vote of thanks to the lecturer.

### AN EXCELLENT EXAMPLE.

In the annual report of the Bath District Nursing Institute it is stated that during last year the Association has been quite apart from the private nurses' home. This is as it should be, and is, of course, in accordance with the conditions of the Jubilee Institute, to which it has been affiliated The nurses now work under a superintendent, who has arranged the city in four districts. During the last 12 months 3,831 visits were paid to 88 patients in the first, 3,647 visits to 99 patients in the second, 2,794 to 81 patients in the third, and 3,712 to 84 patients in the fourth. In addition to these, 2,800 visits were paid to 543 midwifery patients. A successful sale of work has just been held on behalf of this admirably managed organisation.

### EXAMINATION OF HOLBORN INFIRMARY PROBATIONERS.

The Infirmary Committee of the Holborn Guardians have expressed their satisfaction with the report which has been sent to the board by Dr. J. J. Perkins, Assistant Physician, St. Thomas' Hospital, who recently examined 17 nurses who had studied for certificates. The answering of the successful candidates, whose number of marks ranged from 75 to 96, is described in the report as remarkably good, showing that not only have they been well and practically taught, but have been earnest workers and made the most of their opportunities. We agree with the committee that the result reflects the highest credit upon all who are responsible for the training of the probationers at Holborn Infirmary.

### SHORT ITEMS.

On April 4th Sister Edith Maude Pottinger, of the Peak Hospital, Hongkong, daughter of General Pottinger, was married from that institution to Mr. Claud Ivor Williams of the Chinese Imperial Maritime Customs. Miss Pottinger was trained at the Royal Surrey County Hospital, Guildford, after which she went to Northern Nigeria, remaining there for 12 months. Since August, 1904, she has been one of the three sisters at the Peak Hospital, Hongkong.—The second annual meeting of the Rural Midwives Association will be held at 66 Ennismore Gardens, on Thursday, May 18, at 2.45 p.m.—The fête in aid of the Hammersmith and Fulham District Nursing Association at Carnforth Lodge, at which Princess Louise has consented to receive purses, is to be held on Thursday, June 29.

### The Nursing Outlook.

"From magnanimity, all fear above;
From nobler recompense, above applause,
Which owes to man's short outlook all its charm."

### THE PRIVATE NURSE.

Last week we considered why hospital-trained nurses sometimes fail in private cases. In justice, something should be said, in regard to the difficulties and trials, which are peculiar to the work of a nurse, who devotes her life mainly to private cases. Each patient is the member of a separate family, which may or may not constitute a well-regulated household, and a private nurse to be really happy in her work, should be cosmopolitan in her tastes, and possess a power of adaptability not given to all workers. The strain must often be severe owing to the nature of the ailment from which the patient is suffering, but that strain may be immeasurably increased in private cases, where the friends are inconsiderate or ignorant, or worse. The ordinary mistress of a household is naturally upset when severe illness enters the family, and the irritation, which she may suffer in consequence, is not calculated to make her, at first, at any rate, as pleasant to the nurse, as the circumstances should warrant. It is all very well for the public to complain of the private nurse, but it is due to her that every member of a household should realise the difficulties which she has to face, and for their own sakes as well as for hers, they should resolve to meet her half way, to try and anticipate her reasonable requirements, and to so adjust matters, as to make the sick-room arrangements the cause of as little upset in the household, as possible.

It takes many people to make a world, and the private nurse in the course of a year's work enters all sorts of households. For a month perhaps her work may lie in some mansion where the selfishness of riches has made the inmates unconscious of the just claims of a trained nurse. The private nurse, in such a household, is often subject to trials which she ought to be spared, and which she most certainly would be spared, if there was less selfishness and more forethought on the part of her employer. Then, too, character has much to do with the trials of private nurses. A nurse may be an angel, but to be of practical use she must always continue to be a woman too. The more difficult the case of illness, therefore, the less should she be subjected to disabilities which may deplete her energy—disabilities too, that often arise from the faults of the employer, rather than from those of the nurse.

Probably one of the most difficult households, and yet one which a tactful nurse will often find to be in reality the happiest sphere of work, is that where the means are restricted, and so everything has to be done in the simplest and most economical manner. Here the nurse, who is also a woman of the world, will exhibit a bright and cheerful spirit, making the best of everything. With a little practical experience she may soon win the gratitude of the head of such a family, by utilising everything to the best advantage, and so reducing the expenses of illness, as much as possible. It is not, as we said last week, the fault of the private nurse who fails, in a case like this, so much as that of those who are responsible for her training. No nurse who has not great tact, much power of adaptability to circumstances, and a ready way of ingratiating herself with strangers, should attempt private nursing. Without these gifts, and in the absence of practical training, a nurse is disqualified to face the many difficulties incidental to private nursing, for the worries and trials which she experiences, will, in her case, be due, more to her lack of necessary gifts and knowledge, than to any other cause.

The best private nurse and the happiest is probably the woman who has been brought up in a large family, where her angles have been rubbed off, and her general knowledge and tactfulness have been fully developed. We find that the public, in this country, greatly appreciate the services of nurses attached to a sisterhood like that of St. John the Divine, because every member is imbued with a sense of duty and responsibility, which tends to destroy introspection, and to make each nurse sink her individuality and personal likings, for the moment, in the service, she lovingly renders, to the sick person under her charge. So the motherly woman, the mistress of a household who makes it her pride to consider every inmate, however humble, in reality a member of her family who is entitled to consideration, in due proportion, at all times, finds, as she deserves to find, that when the services of a private nurse are needed, those services are rendered, in such a way, as to diminish, and not to increase the anxiety and strain of sickness in the household. Such a mistress realises the risk which all private nurses run, is able to appreciate the difficulties and the nervousness which a nurse may feel on first entering a strange house, and, as she shows consideration, she at once secures the best and readiest service which the nurse can render. Thus both employer and nurse, mutually, though often unconsciously, combine to secure the maximum of comfort for the patient, and the smooth working of everything connected with the sick-room. It follows that the public, who are wise, will ponder these things, and that the private nurse for her part, especially if she is new to her work, will bear them well in mind. Under such conditions the private nurse will find her work, despite its many difficulties, attractive from its infinite variety. It should prove grateful to her feelings, too, from the gratitude which her services will habitually call forth.

### The Nursing of Sick Children.

By James Burnet, M.A., M.B., M.R.C.P.Edin., Registrar, Royal Hospital for Sick Children; Clinical Tutor, Extramural Wards, Royal Infirmary; and Physician to the Marshall Street Dispensary, Edinburgh.

### VI.—VOMITING AND DIARRHŒA CASES.

During the summer and autumn months cases which combine the two symptoms of vomiting and diarrhœa are very commonly met with. Infants are usually the sufferers. The cause is not very well understood even yet, but there can be no doubt that when the atmospheric temperature is high we find the greatest incidence of these attacks. These are often termed summer diarrhœa. The principal cause, however, is found in the food of the infant. Breast-fed infants are comparatively rarely attacked, while bottle-fed babies, on the other hand, often fall victims to this disease. Those, again, who have been suffering recently from gastro-intestinal indigestion are more liable to become affected, and, accordingly, during the warm months, the greatest possible vigilance should be kept over the infant's food supplies.

Organisms are always present in the alimentary tract, but when the latter is irritated by the giving of improper food its lining membrane becomes impaired, and hence these organisms obtain a ready entrance. They go on multiplying rapidly, and, during their growth, they manufacture a poisonous substance, which is taken up by the blood, producing a kind of general intoxication of the whole body.

The symptoms of this affection, apart from the vomiting and diarrhœa, form a very definite group. At first the infant is restless and refuses all food. His thirst is great and the temperature rises rapidly to 103° or even to 105° F. The vomiting makes its appearance first. At the start the food is vomited, and then the vomited material consists of bile-stained mucus. The legs are often seen to be drawn up, and the abdomen is hard. Presently the bowels are evacuated, and the pain in the abdomen abates. Within a very short interval another, and still another, evacuation takes place. The motions rapidly lose their natural colour and become greenish, their odour being very disagreeable. The vomiting continues, any attempt at feeding being followed almost immediately by vomiting and purging. It is quite a common occurrence to have as many as 15 or even 20 motions in a day.

The eyes become sunken, and usually we find dark areolæ under the lower lids. The fontanelle is depressed, and the face, together with the body generally, assumes a deadly pallor. The hands and feet are cold, and the temperature often becomes subnormal, while the pulse is apt to be very soft and feeble in character. If the case is not promptly placed under treatment the infant speedily becomes greatly emaciated, and marked symptoms of collapse set in, associated with very feeble respiration and a pulse which is hardly, if at all, perceptible. Convulsions are not infrequently met with in such cases, while a chronic form of gastro-enteritis may be induced should the symptoms of vomiting and diarrhœa not yield readily to treatment. Broncho-pneumonia may set in as a complication, while occasionally the motions will be found to be tinged with blood, owing to excessive irritation of the lining membrane of the intestinal tract.

The first step in the management of such cases is to withhold all food entirely for 48 hours. This gives the stomach and bowels time to recover. The next thing to do is to wash out the bowel with a solution of boracic acid. The strength used should be about 30 grains to the pint. This should be used to flush out the bowels, and the injection should be continued until the solution is returned perfectly clear. These injections not only soothe the irritated lining membrane, but they also tend to stimulate the patient. In very bad cases the stomach should also be washed out with a similar solution. The medical attendant performs this operation himself, but the nurse should know how it is done, as she must have everything in readiness beforehand. The solution should be placed in a pint jug. A mackintosh must be at hand to pin round the infant during the process. A little glycerine should be placed in a wide-necked bottle. This is used for lubricating the tube before passing it into the gullet. A small basin will also be required to catch the washings as these are returned from the stomach. After the process of washing out the stomach has been completed it is the nurse's duty to cleanse the tube, and this is most readily done by allowing water from a hot tap to run through it several times. It is then to be placed for a quarter of an hour in a basin containing boracic lotion, and finally dried.

Although all food must be withheld for forty-eight hours the child may be allowed sips of barley water or of albumen water at frequent intervals. The latter is made by taking the white of an egg and mixing it thoroughly with three-quarters of a pint of water with a little salt added. Plain boiled water, however, serves equally well, as the infant is not so much in need of nourishment as of stimulation.

The cold extremities afford a valuable indication for treatment in these cases. The infant must be kept warm. This may be accomplished by rolling him up in cotton-wool, and then encircling him in a couple of blankets, outside of which hot-water bottles may be arranged. Great care must be taken that the blankets intervene between the patient and the bottles, otherwise serious injury may be caused. A hot mustard bath is also a useful means of stimulating such cases, and when the respiration and pulse become feeble this should be resorted to. A few drops of good pale brandy in a teaspoonful of water will often be found serviceable as a stimulant.

When the vomiting and diarrhœa have somewhat abated the infant must be fed with great care. Small amounts should be given every hour or hour and a half. I am very fond of starting with two tablespoonfuls of whey mixed with an equal quantity of barley-water and a small quantity (say, half a teaspoonful) of sugar of milk added. These feeds should be given iced by preference. Instead of this sherry whey may be given. It is made by adding a wineglassful of good sherry to half a pint of fresh milk. This is brought to the boiling-point and then strained. The resulting liquor is very pleasant to take and has the additional advantage of being a good stimulant.

The ordinary feeding of the patient must be very gradually resumed. I find that it is often best to begin with whey mixtures containing cream in the following proportions:—Whey, 2 tablespoonfuls; barley-water, 2½ tablespoonfuls; cream, a dessert-spoonful. This mixture may be given every two hours. After this ordinary mixtures of milk and barley-water may usually be safely given, but care should be taken not to make them too strong at first.

The nurse must never forget that the stools of infants suffering from summer diarrhœa are infectious. All soiled napkins should accordingly be instantly removed from the patient and from the nursery and placed in a hot antiseptic solution, such as a 1 in 1,000 corrosive or a 1 in 40 carbolic. They are then to be boiled for some hours and finally washed in the ordinary way. The buttocks are apt to become red and excoriated from the passage over them of the irritating evacuations. Accordingly the nurse should carefully wash the parts after each motion with warm boracic lotion and then dry thoroughly with a piece of absorbent wool. Finally, she should dust on a little violet or starch powder.

I have said nothing about medicines, as the prescribing of these must be left to the physician in charge of the case. He will sometimes order a preliminary dose of castor oil, and usually a tea-spoonful is enough. This is often ordered instead of the washing out of the bowel already referred to, and which I consider decidedly preferable. During convalescence the nurse should see that the infant is warmly clothed, as, being considerably emaciated, he will very readily take cold. It is of special importance that the abdomen and legs should be well covered, preferably by a close-fitting knitted garment consisting of drawers and gaiters in one.

### The Nurses' Clinic.

### THE DISTRICT NURSE IN RELATION TO THE AGED POOR. BY A SUPERINTENDENT OF DISTRICT NURSES.

Many aged people infinitely prefer living alone to entering the most comfortable of modern workhouses, and they are perfectly capable of doing so if only they have the district nurse to rely upon in case of acute illness, or to attend at regular intervals to give any assistance demanded by a chronic complaint. The average cost of the visits paid is less than sixpence, and there are many cases which we do not attend, on an average, more than 10 times a year. Let ratepayers consider what this means.

The nurse should always be on good terms with the relieving officer, as in cases of neglect, or suspected ill-treatment of the aged poor, he is often a kind and most practical adviser. It not infrequently happens that old people who have outlived all their near relatives, but who are possessed of savings, or a small pension, fall into very bad hands, and are as much in need of deliverance from their false friends as if they had been captured by brigands. I have known instances where elderly women in this position have been attended for a time by younger women with apparent disinterestedness—or at any rate have received fair market value for their money. Presently a husband is introduced; very soon the home seems to belong to the married couple, and before long the nurse finds the door closed in her face.

On the other hand, many a landlady, in return for incredibly small payment, is daughter, servant, and nurse in one to men and women with no claims upon her except age, helplessness, and the still more indefinitely binding one of being "that respectable."

Poor people marry so much younger than the middle classes, and death and separation play such havoc among their children, that often from the age of 50 or 55 they are as much alone in the world as if their family had never existed. And only the best of the poor, the best intellectually as well as morally, are as yet civilised enough to care very deeply for their parents after they themselves have reached maturity. Among the educated classes one hears people say, "I do not think that I truly loved or appreciated my parents until my own children were born;" but where the struggle for bare existence is keener, the growth of new ties means the slackening of the old. Children whose grandparents are in the workhouse—or half starving themselves rather than go there—are to be seen in every street elaborately dressed and expensively, if insufficiently, fed. In many houses where there is a parlour, or even two, there is "no room" for the aged widow or widower.

There are, of course, numberless cases where the old people are allowed to share the home of married children, and the difficulties of this arrangement can be considerably smoothed by a tactful district nurse. The intercourse between the old and the young is often embittered by a constant irritability and fault-finding on the part of the former, and principally for want of certain attentions which the latter lack time or patience to perform. A bi-weekly visit from the nurse, and the comfort of "a good wash" without the risk of catching cold, often make a great improvement in health and temper, and as "spite breeds hate, and kindness friends, and patience peace," the whole condition of the home is improved.

In all ordinary cases where loveless young and old are living together, the nurse can be of great use as a safety valve if she will merely listen to the complaints on both sides, do very little, and say still less. After ten minutes of bitter complaints poured out on the doorstep or in the back yard, a daughter or daughter-in-law often returns to her duties so much relieved by "having had her say," that she can manage to be kinder for a week after; while the old people having told the nurse long tales of her fecklessness, extravagance, or over-indulgence of her children, are content to store up a fresh list of grievances silently in preparation for the next visit.

The nurse must not imagine that every old person who grumbles is necessarily in unkind or even unsympathetic surroundings. A certain amount of grumbling is a healthy, or at least a normal, condition, just as occasional wilfulness may be in children. The signs of harsh treatment or cold neglect are rather to be found in an unnatural timidity, quietude, and reticence.

The aged poor, although they commonly retain to a late age the power of eating coarse provisions, such as bacon, cheese, and cabbage, nevertheless frequently suffer for want of equally cheap but more suitable food. I have known old people driven to live on bread and butter and tea six days in the week, not because of the poverty of the household, but because they could not get their teeth through cold meat, and no vegetables were cooked until the son returned from work at half-past six or seven, an hour when they could not

THE NURSES' CLINIC—*Continued.*

accustom themselves to eat. There was no valid reason why a milk pudding, vegetables, or soup should not have been prepared for their midday meal, and it would have been a benefit to the children of the house as well as themselves if these things had been provided. The nurse must do what she can to improve their usual diet, and perhaps the suggestion that they must have a milk pudding or a boiled egg every day when there is no hot meat or soup is the simplest and the most likely to be carried into effect. In the vast majority of English working-class homes it is not poverty that prevents young and old from being properly fed, but ignorance, laziness, and apathy. It is true that even the most elementary cooking takes time and trouble, but when a woman has no work outside her tiny house, and her children from the age of three are "minded" by the State for five hours a day, she ought not to lack leisure and energy. The cooking-stove is, unhappily, very poor in many houses, but I have seen none so bad that they could not at any rate boil a saucepan, toast a piece of meat, or bake a milk pudding.

Comfortable house-boots are greatly needed by the aged poor, and one-half of the shawls and petticoats bestowed upon them would be gladly exchanged for felt boots and shoes, waistcoats, kneecaps, mittens, loose nightcaps, warm smoking-caps, hassocks, soft pillows, and hot-water bottles.

The old-fashioned poor suffer greatly from boredom and low spirits when no longer able to do hard work, and the nurse should try to provide some suitable employment for them, the furtherance of any for which they show a preference being of course more easily practicable than the introduction of anything new. It is generally possible for her to find some genuinely charitable person willing to provide the necessary materials.

When people are urged to establish district nurses in their town, or to support those already there, one hears most of acute illnesses, and of the lives lost for want of skilled attendance. As a matter of fact, it is chiefly for agonising or exhausting chronic complaints, for small injuries that may so easily be aggravated into lifelong disabilities, as a practical teacher of hygiene, as a preventer and forestaller of illness of every kind, and as a friend and protector of children and of the almost equally helpless aged poor that the district nurse is needed.

### A Professional Visit to Tangier.

BY A SISTER.

In my professional capacity of nursing sister I was asked to accompany a doctor to Tangier in order to bring home a patient who was supposed to be very seriously ill. We left Tilbury in a P. and O. steamer on a fine morning and had a capital voyage through the Bay of Biscay and along the coasts of Spain and Portugal until we came to the Straits of Gibraltar and in sight of the famous Rock. Here we had to wait two days for the Tangier boat, when I made the most of the time by seeing as much as possible of the little fortress town. I drove through the Alameda Gardens towards Europa Point where the Governor's cottage is situated and also the new military hospital, walked in the Spanish and Moorish markets, seeing the respective natives in their picturesque dress with their various commodities, went through the rock galleries which are now only occupied by obsolete guns, saw the Moorish palace used as a civilian prison, and finished up by looking at the shops, which are numerous and full of quaint wares. On the second day the doctor kindly took me to Algeciras, half an hour by boat from Gibraltar—a small Spanish town remarkable only for its ill-paved streets—and an Alhambra where bull-fights are held. We had lunch at a charming hotel, the Reina Cristina, which is built in Spanish style with a tiled patio in the centre filled with lounge chairs, plants, and a fountain, and into which all rooms open, the building being of one storey. It has an extensive garden and is in a magnificent position overlooking the Mediterranean with Gibraltar and the coast of Africa to be seen in the distance. The next day we sailed for Tangier and reached there in about four hours in rough weather, which made the landing in small boats look rather formidable.

### The Patient Convalescent.

However, in company with the British Consul and his wife, we arrived safely on the small wooden pier and found ourselves in the midst of a crowd of natives of every degree of colour in their dusky robes and turbans, and all shouting and gesticulating wildly. A stately Moor, sent to meet the boat, rescued us and our luggage in a marvellous way, and we walked through the town, calling at the Hôtel Ville de France, on our way to the Villa Senya el Nashti (the garden of wells), where the patient resided. To my surprise, a district nurse escorted us, who explained that the worst of the illness was over and the patient convalescent. How am I to describe the beauty of the garden by which the villa was surrounded? In it were palms and tropical trees of every description, with flowers in profusion; plumbago and heliotrope climbing wild in the trees, scarlet poinsettias, camelias, begonias, violets, geraniums, and arum lilies, besides a multitude of fruit trees; mandarin oranges, bananas, and pinepapples, guavas, quinces, custard apples, and others. The next day I was taken for a mountain ride on a donkey, my first experience of a pack-saddle, accompanied by native boys, who have marvellous dexterity in holding one on, up and down the most precipitous tracts, which I cannot dignify by the name of roads.

### District Nursing.

The district nurse also took me into some native huts, which are built of mud and straw, and consist of a single room with mud floor, and without furniture of any description. The sick folk simply lay on rugs on the floor, looking pictures of misery. It was very uphill work nursing under these conditions, generally without even a doctor's advice, the nurse having to rely on her own judgment as to remedies, and to do what she could for the relief of her poor patients. She could not be grateful enough to the London doctor for his kindness in seeing several of the worst cases during his short stay. Later, I had pointed out, a small hospital, which is worked by members of a medical missionary society. Sunday is market day when the country people come in to the great square outside the Socco Gate, with their wares consisting chiefly of fruit, vegetables, and charcoal. Caravans and camels were absent, to my disappointment, as owing to the disturbed state of the country it was unsafe to travel, but the streets were thronged with a motley crew, Moors, Spaniards, and Portuguese, mules and donkeys mixed indiscriminately. The main street where are the bazaars, is most badly paved with cobblestones, and on rainy days the dirt is simply indescribable. In company with the doctor and a guide, I was taken to see the prison and courthouses—the outside only of several mosques, and a saint's or priest's house which is sanctuary for anyone having committed murder. I was shown inside a harem where were Moorish beauties much painted and powdered. After visiting the old walled part of the town with its antique Spanish fortifications, narrow lanes, archways and houses, the whole being a most interesting and picturesque survival of mediæval Orientalism, we proceeded to the English church. This is a small building with Moorish arches and Arabic inscriptions, erected chiefly through the exertions of the late Sir John Drummond Hay, who was for forty years British Minister in Tangier. In the villa where I stayed, the Moorish and Spanish servants were worth notice. The housemaids wore white loose garments with an elaborate belt or sash which served as a pocket and hiding place for charms to protect them from the evil eye of the Christian. The butler was a most dignified looking rascal who rejoiced in the name of Ahmed el Gharbie, and who salaamed and left his shoes on the threshhold, entering the room barefoot. He also wore white flowing robes and a white turban, the latter signifying that he was a married man. The day of our return the patient and I left the villa, after most pathetic farewells from the native servants—men, women, and children—and proceeded on donkeys through the town to the quay, in a tremendous downpour of rain, when to hold an umbrella was almost impossible, and to have fallen off the animal's back would have been to render one's self unrecognisable from filth and mud which lay in pools all along our route. Our goods accompanied us on mules in panniers, and truly thankful I was to find myself once more safely on board the boat with doctor and patient, on my way back to Gibraltar, *en route* for London.

### Practical Hints.

We welcome notes on practical points from nurses.

### THE USE OF WHITE ART MUSLIN.

DISTRICT nurses may be glad to know that an appreciable difference may be made in the yearly "nursing requisite" bill by the use of white art muslin in many ways, instead of more expensive materials. The coarsest make is the best, and it can be bought at 10½d. per dozen yards from any large draper's shop. Cut into small squares it makes an excellent substitute for wool for cleansing mouths.

Pneumonia jackets can be made equally well with muslin and absorbent wool as with gamgee tissue and at considerably less cost.

Sterilised and sprinkled with iodoform powder the muslin makes a good substitute for iodoform gauze. Nurses who have cases of carcinoma or septic wounds calling for frequent dressing, for which they may be using iodoform gauze, will find this a real boon, since they need not worry about the cost so much. Of course, the iodoform must be used very sparingly, remembering the consequences of its too great absorption.

The muslin may be used as handkerchiefs in cases of phthisis, diphtheria, etc., where the ordinary handkerchief which cannot be burned is dangerous, because of risk of infection to others.

A good foundation for poultices may be made of muslin when "old rag" is at a premium and has the advantage of being light, therefore lessening the weight of the poultice.

### Where to Go.

ANNIVERSARY meeting of the Society for the Propagation of the Gospel in Foreign Parts. Meeting in connection with Women's Work at Church House, Tuesday, May 16th, 3 P.M.

### Messrs. Macmillan's New Venture.

### "HOW ARE THE MIGHTY FALLEN!"

MESSRS. MACMILLAN AND CO. state in their Preface to the first number of the *Nursing Times*, issued by them on the 6th instant:

> "We may well begin by stating, in order to prevent any misconception, that this journal was started solely by the publishers (*i.e.* by Messrs. Macmillan), and that it is their property, and theirs only."

Much of the first number, however, proves on examination to be, in fact, not the property of Messrs. Macmillan but the property of THE HOSPITAL.

Thus, the whole framework, and the size and colour of the paper of Macmillan's journal are so similar to as to appear to be identical with the *Nursing Section* of THE HOSPITAL. Further, the arrangement of the advertisements, their type, classification, headings, and much of their matter, and even the wording of the business announcements which appear, have been followed from THE HOSPITAL, with a faithfulness of imitation, which is the sincerest of flattery. Many of our advertisers will be aware that, for some time past, Messrs. Macmillan have offered free insertion of the advertisements, cut from THE HOSPITAL, for a number of consecutive weeks. Macmillan's first number, therefore, contains numerous advertisements, which must be stale matter for the reader.

Messrs. Macmillan are new to the publication of a journal for nurses, or they would never have thought it a kindness, to invite such hardworking women, when seeking employment, to apply for posts which were probably filled up before the advertisement appeared in Macmillan's venture.

Again, Messrs. Macmillan publish *over a column of appointments the majority of which are lifted, word for word, from back numbers of* THE HOSPITAL.

Yet Messrs. Macmillan claim that their journal, made up in this way, is "of good tone and representative of all the best elements in the nursing world." When judged by Messrs. Macmillan's practice, as here revealed, this statement may be taken as exhibiting the low opinion Messrs. Macmillan must hold of the morals and honesty of the readers they intend to cater for. Such readers, they appear to think, will have an insatiable appetite for these stale advertisements, this stale and "lifted" news, as the first number of their journal shows.

In any case, these proceedings of Messrs. Macmillan, if not called by a harder name, certainly amount to such an annexation and utilisation of the ideas and methods of other persons, as to be widely at variance with the best traditions of British journalism. It will be observed, that Messrs. Macmillan proudly claim, that they alone are responsible for this utilisation and annexation of the ideas and methods of other people. Well, therefore, may it be proclaimed "How are the mighty fallen!"

### To Nurses.

WE invite contributions from any of our readers, and shall be glad to pay for "Notes on News from the Nursing World," or for articles describing nursing experiences at home or abroad dealing with any nursing question from an original point of view according to length. The minimum payment is 5s. Contributions on topical subjects are specially welcome. Notices of appointments, letters, entertainments, presentations, and deaths are not paid for, but we are always glad to receive them. All rejected manuscripts are returned in due course, and all payments for manuscripts used are made as early as possible after the beginning of each quarter.

### William Rathbone: the Founder of District Nursing.

The fifth chapter of the admirable "Memoir of William Rathbone" (Macmillan and Co., Limited), by his daughter, Miss Eleanor F. Rathbone, is devoted to a review of his work for nursing, and it is appropriately headed with a quotation on the subject from "Life and Labour" by Mr. Rathbone's old friend Charles Booth. It was after the death of his first wife in 1859 that William Rathbone began to interest himself in nursing, owing to the fact that she was attended in her last illness by a nurse whose skill had done much to ease her. As his daughter says:—"Seeing how much difference trained nursing could make, even in a home where every comfort and appliance that affection could suggest was provided, William Rathbone began to think what illness must mean in the homes of the poor, where comforts, appliances and skill were alike wanting." He, therefore, resolved to try an experiment, and asked Nurse Robinson to engage herself to him for three months to nurse poor patients in their own homes in a certain district of Liverpool. She was provided with the most necessary appliances, and arrangements were made for supplying such nourishment and medical comforts as might be required to make her nursing effective. At the end of a month she came to her employer in tears, and asked to be released from the remainder of her engagement because, as she told him, the amount of misery she had to see was more than she could bear. With some difficulty he persuaded her to go on, with the result that at the end of the term she declared that if Mr. Rathbone would keep her on she would never go back to any other kind of nursing. The good she found herself able to do and the gratitude of her poor patients had quite reconciled her to the sadness of her work. Mr. Rathbone himself confessed that its effects seemed greater than even he had expected. "Lives had been saved which, without the nurse's care, would almost certainly been lost; patients considered chronic had been restored to health; object lessons in the value of cleanliness, order, and fresh air had been given in many homes." In addition to this—as Mr. Rathbone's own book "The History and Progress of District Nursing" shows—"the nurse had helped to prevent the moral ruin, the recklessness, the drunkenness, and the crime which so often follow upon hopeless misery. Within the space of a few months she had had two cases in which the wife's sickness had thrown a household into disorder, and the husband, unable to face the wretchedness he knew not how to remedy, had taken to drink. The nurse showed what might be done to restore order and to lessen suffering. The husbands, who were well-meaning, industrious men, took heart again, left off drinking, and were saved, together with their families, from the state of utter wretchedness and collapse towards which they were fast drifting when the nurse came to their rescue."

This was the beginning of district nursing as understood in the present day, and Mr. Rathbone, considering the success of the experiment was proved, began looking about for suitable nurses in order to extend it. The difficulties he experienced in finding them were so great that he very wisely determined to consult Miss Florence Nightingale, who suggested that Liverpool had better form a school to train nurses for itself in its own hospital. At that time the Committee of the Royal Infirmary (then the principal Liverpool hospital) were endeavouring to attract a better class of women to the work by offering increased salaries, but the matron had found only four whom she could trust not to celebrate such a rise in prosperity by getting drunk on quarter-day. Hearing that Mr. Rathbone was devoting his attention to the improvement of nursing, the committee invited him to join them, and having consented, he shortly afterwards undertook to build a training school and home for nurses and to present it to the Infirmary. His daughter observes that while the planning and building of the Home cost him much time and thought, the work brought him one great personal pleasure. "It was the beginning of an intimate friendship with Miss Florence Nightingale, upon whose advice he depended chiefly in the preparation of the plans, and who gave to them, as he said afterwards, as much attention as if she were herself going to be the superintendent of the School." The first superintendent was Miss Merryweather, who had undergone a short training at St. Thomas's Hospital, and on May 1st, 1863, the new Home was formally opened.

Meanwhile, the work of district nursing had been making rapid progress, and by the end of 1863, or six years after Nurse Robinson had first gone into the homes of the poor, the whole of Liverpool was divided into eighteen districts, each district being placed under the care of a nurse and of a group of ladies, who undertook to superintend her work, to pay for her lodging, and to provide the nourishment and medical comforts needed for patients too poor to provide them for themselves. Mr. Rathbone was the first honorary secretary of the new training school, and at an early stage of its history took the place of one of the lady superintendents during her absence for about a year, going with the nurse weekly to visit her patients. The experience thus gained induced him to take up reform in another branch of nursing, and, as our readers are well aware, Mr. Rathbone subsequently devoted a great deal of his time and abilities to the task of elevating the standard of nursing in workhouses and infirmaries. It was due to his efforts that, in May 1865, the wards of Brownlow Hill Infirmary were placed under the charge of Miss Agnes Jones, with twelve nurses from St. Thomas's Hospital and eighteen probationers. Some exceedingly interesting particulars are given by Miss Rathbone respecting the engagement by Mr. Rathbone of Miss Jones, which had the most happy results; but, unfortunately, before the third year was completed, she fell a victim to an attack of typhus fever. How much Mr. Rathbone appreciated her services during those three years may be gathered from the statement that his achievement in finding her and bringing her to Liverpool was the one fact upon which he was accustomed to dwell with pride as well as with satisfaction.

For the remainder of his life, however, Mr. Rathbone warmly interested himself in various developments of nursing. When he was one of the members of Parliament for Liverpool he acted as chairman of the organisation which was formed to start a district nursing association in London, Miss Florence Lees—now Mrs. Dacre Craven—being one of the honorary secretaries. In 1887 he was consulted by the trustees of Queen Victoria's Jubilee Fund, and when the trustees transferred the executive part of their duties to a provisional committee, he undertook to act as honorary secretary. Here he was entirely in his element, and when the committee met for the last time in November 1889, they placed on record, "their deep indebtedness to Mr. Rathbone for his unceasing and most valuable labours." The Queen appointed him vice-president of the council, and at the earnest wish of his colleagues he retained that post until his death. In summing up his characteristics Miss Rathbone pays a just tribute to his detailed knowledge of a nurse's profession, and his wide and lofty conception of its scope and aims. But, "although he had a sympathetic appreciation of their professional point of view, he never let them forget that their work was a vocation as well as a profession, and that they must be prepared, if necessary, to sacrifice their personal ambition to their usefulness." He was a pioneer, indeed, who combined, in a remarkable manner, the spirit of the enthusiast with the sound judgment of the man of affairs.

### The Incorporated Society for Promoting the Higher Education and Training of Nurses.

### ITS OPPONENTS' OBJECTIONS, AND THE SOCIETY'S ANSWER TO THEM.

**A meeting was held in the deputation-room of the Board of Trade, 7 Whitehall Gardens, S.W., on Friday, the 5th inst., Mr. George S. Barnes, comptroller of the Companies Department, in the chair. The meeting had been called by Mr. Barnes by a letter dated April 10th last, to hear objections in opposition to the granting of a license to the above-named society to incorporate without the word limited after its name, at which the Board of Trade had expressed the wish to hear the objectors personally, and not solicitors or counsel on their behalf. Amongst those present were Sir Victor Horsley, F.R.S., Mr. Andrew Clark, D.Sc., Dr. Langley Browne, Dr. Galton representing the British Medical Association, Sir James Crichton Browne, Dr. Bezley Thorne, Dr. Cummings, Dr. Barclay and Mr. Langton representing the Royal British Nurses' Association, Mr. Charles Burt and Mr. Sydney Holland, the Central Hospital Council, Mr. H. R. Swanzy, vice-president, representing the Royal College of Surgeons, Ireland, Miss Isla Stewart the Matrons' Council, Mrs. Bedford Fenwick the State Registration Society, Dr. Bedford Fenwick, a nurse representative of the nurses in Ireland, Mr. Cosmo O. Bonsor, Miss Swift, Miss Catherine J. Wood, Miss Breay, and several others. The objectors were nvited, in turn, to state the grounds of their objections, which were taken down in shorthand for the information of the President of the Board of Trade, and Mr. Cosmo Bonsor was then asked to reply, as rep-esenting the Incorporated Society for the Higher Education of Nurses.**

### THE OBJECTORS' CASE.

The objectors' case has been set out in various petitions, and in statements published in the Press during the ast few months. The objectors may be classified under three heads:—(1) The Royal British Nurses' Association charged with the voluntary registration of nurses, and the advocates of State registration; (2) the opponents of State registration, and several of the nurse-training schools as represented by the Central Hospital Council for London; and (3) the advocates of entire freedom for matrons and nurses, such freedom to include entire independence for both from any influence which may at present be exercised over them by the hospital and institutional authorities, and those who may generally be classed as employers.

#### 1. The Royal British Nurses' Association and the State Registrationists.

Although each of these parties is promoting a separate Bill in Parliament for the State Registration of Nurses, they agree in opposing the Incorporated Society for Promoting the Higher Education of Nurses. They contend, for example, that no such society should be incorporated with the authority of the Board of Trade or otherwise as a voluntary association, pending the passing of an Act of Parliament to secure the State Registration of Nurses. That to incorporate the Society to-day must delay State registration, and that when the latter is secured the Incorporated Society must necessarily degenerate into an enfeebled and useless body. Assuming that State registration is not granted, and that the Incorporated Society is organised and succeeds, it is further contended that this success would call forth a society for the higher education of nurses in Wales, in Scotland, and in Ireland, with the result that the present confusion in nursing affairs must increase. That if the Incorporated Society is not opposed to State registration, its scheme should be handed over to the Royal British Nurses' Association which already occupies the ground and has 2,800 members of whom 2,600 are on its register. That in self-defence the Royal British Nurses' Association must oppose the Incorporated Society whether it is likely to prove good or bad in its effects upon nursing generally. That, clothed in the verbiage of the Stock Exchange, the society is an attempt to capture the examination and registration of nurses by a body of laymen who have no claim to interfere with the affairs of nurses. That a nurse who had passed the society's examinations and was entered on its register might have her name struck off by laymen, which would be a great injustice to herself whatever her conduct may have been, seeing, as the objectors contend, that all the council might be laymen. It is essential, the Royal British Nurses' Association opposers contend, that medical men must have a paramount, or at least a predominant voice in nursing affairs. The Royal British Nurses' Association enforces discipline by striking off its register nurses who may be guilty of misconduct or intemperance.

So far as Ireland is concerned, the promoters of the Society have not, it is stated, consulted Irish opinion, and Ireland is opposed to its incorporation. Irish nurses object to have nurses reduced to a commercial level, and to the setting up of a London monopoly. The State alone can properly register nurses, and State registration must be controlled by a body directly representative of all nurses throughout the country. Any philanthropic or voluntary scheme must be objectionable, and, to possess the necessary authority, State registration must be representative. The Bills before Parliament recognise this principle. All volunary efforts at registration have failed and must continue to fail. Private people cannot group themselves together to carry out a public purpose which the State alone can properly undertake. The medical profession as a whole are in favour of the principle of the registration of nurses, providing the medical profession are given the power to recognise the qualifications of nurses. They cannot, however, recognise pseudo registration, but only State registration. Finally, that a State guarantee of the training of each nurse is called for, which any Act of Parliament granting the State registration of nurses must secure.

#### (2) The Opponents of Registration and the Central Hospital Council.

These objectors have memorialised the Board of Trade against the Incorporated Society on the grounds that it is not promoted by the majority of the nurse-training schools, but by a minority who have thus exhibited a hardihood which is not appreciated by those who are not promoting the present scheme. That some of the great hospitals of London consider the present proposal to be very undesirable, and they are opposed to the granting of a license by the Board of Trade. That the Central Hospital Council is against the registration of nurses altogether, but it is working in the direction of uniformity in curriculum for nurses. These objectors ask the Board of Trade to deal at once with the application of the Incorporated Society, and not to delay their decision as some of the other objectors suggest. That

### THE INCORPORATED SOCIETY—*Continued.*

the fact that the Board of Trade refused the licence to the Royal British Nurses' Association in 1891 is a good reason why the same course should be followed in 1905. That in effect the Incorporated Society will promote registration, and as the House of Lords Committee decided against a proposal of the kind, it shows great temerity for a few schools to promote an incorporated society with this in view, as they are at present doing. That all registration must fail, and that State registration would be the worse of two evils. That the Incorporated Society is promoted by gentlemen who bear the greatest names in London, but they practically know little or nothing about nursing. That the scheme owes its present position to the great personal influence of one man, Mr. Cosmo Bonsor, whose agreeable personality is irresistible. That so far as the evidence is at present forthcoming, the Incorporated Society will not have the support of representative matrons of large hospitals. That as these objectors are opposed to all registration for nurses, they are also opposed to the Incorporated Society for the higher education of nurses

3. The Advocates of Complete Independence.

These objectors urge that all nurses are entitled to direct representation upon any organisation which is incorporated for the purpose of dealing with nursing affairs. That nurses must have the paramount voice in the conduct of their business; that nurses have proved themselves competent and capable of doing so, and in future they must be entirely free from all outside control in such matters; that the Central Hospital Council for London is a non-representative body so far as nurses are concerned; that it has petitioned against State registration, but that petition should not carry weight, for the Central Hospital Council is so constituted as to give it no authority to speak for nurses on a matter which primarily affects themselves. They protest against this Council's action as a non-representative body, and the opinions of the matrons of the hospitals represented in its memorial cannot be regarded as independent and personal, seeing that the matrons' employers first passed resolutions against registration, and so restrained their officers from cting independently; that in the same way the ulterior effect of the action of the millionaires who are promoting the Incorporated Society cannot prove beneficial to nurses. That these millionaires contribute great sums to the hospitals; that they are very influential on the councils of the King's, the Hospital Sunday and Saturday and the Nurses' Pension Funds, and that it is preposterous to urge that if once the Society for Promoting the Higher Education of Nurses is incorporated, the nurses can go against them. That in such a case the nurses could not be independent, and that the influences brought to bear upon nurses would be wholly wrong. That nurses should themselves have the direction and control of all moneys obtained from or appertaining to nurses. These objectors state further, quite frankly, that if, as nurses, they are going to obtain educational advantages through the instrumentality of these millionaires, they would rather be without them.

### THE SOCIETY'S ANSWER TO THE ABOVE OBJECTIONS.

That much of the opposition is due to misapprehension. This arises from the publication of an incomplete draft of the memorandum and articles of association of the Society, and that the complete scheme as laid before the Board of Trade is not open to many of the objections raised. That the seven signatories to the articles of association frankly admit that the majority of them know little about nursing, but they are deeply interested in nurses and hospitals. That as men of business they are absolutely competent to mind their own business, and not to interfere with other matters than those they understand. That they have approached the Board of Trade in support of the incorporation of the Society for the Higher Education of Nurses, because they were urged to do so by many nurses, amongst them some of the leading women in the nursing world, and that the scheme of the Society has been well thought out and provides, in fact, not only that nurses will have an adequate voice in the management of its affairs, but that the doctors on the Council of the Society will be selected by the nur.es themselves. That there is no immediate prospect, and in view of the present condition of nursing affairs in this country there is indeed small probability, that Parliament can properly institute State registration for some years to come. That should State registration be set up by Act of Parliament the Society can turn over its organisation to the State authority. That the nurses are entitled to fair play wherever they may have been trained, and that the Society for the Higher Education of Nurses will secure this result for all nurses by its examinations and by its protection of the competent. That it is not opportune at the moment to go to Parliament for State registration, but the machinery of the incorporated Society worked by nurses, with the assistance of members of the medical profession, will pave the way for a thorough organisation of nursing matters in Great Britain, upon a basis which must secure justice and efficiency all round. That there is at present no uniformity of training, and such uniformity is likely to be hastened by the incorporation of this Society. That the Society will be absolutely voluntary; if it is not wanted it will fail; if it is wanted it will succeed. That when the Council of the Society is constituted and the names are published, nurses will be able to judge for themselves whether they will join and support it or not. That the fact that the proposed incorporation of this Society has created such a stir in nursing circles, proves that its objects must, at any rate, include many proposals which the majority of nurses desire to see carried out. That in order to enable such a society to be incorporated, it is essential as a preliminary, that the seven gentlemen of the City of London should apply for incorporation, but once incorporated its affairs will be conducted and controlled by the nurses and the medical practitioners who constitute its council. That if the scheme succeeds, as the opposition it has called forth seems to indicate that it may succeed, the signatories will be more than rewarded for the public spirit they have displayed in coming forward at this juncture to help the nurses to secure many necessary changes in the present state of nursing. That the many evils which this society will remove have tended to confuse the public mind, to create injustices from which nurses should be free, and to impede the raising of the standard of training to a uniform basis of efficiency; and that the Incorporated Society will make it possible for all competent nurses to secure recognition and protection, because the Society will soon place the public and the medical profession in a position to know the true from the false.

### A COMMON-SENSE CONCLUSION.

So far as the objections raised are concerned, it will be obvious to those who understand most about nursing questions, that many of them are self-destructive, and that such arguments as do not come under this category will be found not to apply, in practice, to the scheme of the Incorporated Society for Promoting the Higher Education of Trained Nurses.

### Everybody's Opinion.

[Correspondence on all subjects is invited, but we cannot in any way be responsible for the opinions expressed by our correspondents. No communication can be entertained if the name and address of the correspondent are not given as a guarantee of good faith, but not necessarily for publication. All correspondents should write on one side of the paper only.]

### THE REGISTRATION OF NURSES' UNIFORM.

Miss Dorothy A. Davey, a registered midwife, writes: The recent correspondence in your paper has brought me to the point of suggesting a plan which I have long wanted to publish—namely, a scheme for the registration and protection of nurses' uniform. I am sending simultaneously with this, copies of the scheme to her Majesty the Queen, Mr. R. C. Munro-Ferguson, M.P., Dr. McVail, and others. The following is the scheme which I think might easily be worked :—Each certificated nurse to wear a badge. In order to particularise between, and popularise with, all grades of nurses, it is obviously necessary to have different badges. The badge should be like accompanying sketch—*i.e.*, a silver band (bronze, in the case of fever nurses), with enamel cross and spaces in centre. The enamel cross should be varied in colour. Red for four and three years certificated nurses, blue for two years, green for one year, white for midwives, and a white St. Andrew's Cross for obstetric nurses. On the reverse of the ring must be the owner's name. The medals must be supplied by Government, and nurses ought only to be able to obtain them by producing their certificates. The possibility of such a registration has lately been exemplified by the proceedings at the meetings of the Central Midwives Board. If this could only be done it would obviate the necessity for any other registration of nurses or uniform, as the public could tell at a glance whether or not the owner of the uniform has earned the right to wear it.

### THE ABUSE OF A NURSE'S UNIFORM.

"Beaumont Street" writes: Will you kindly permit one who has been a resident for 30 years in Marylebone to contribute a few remarks on the subject of a nurse's uniform and the abuse of the latter? My long residence in this parish enables me to affirm that the nursing home nuisance has done much to aggravate practices which have brought discredit on the nursing profession. I can well remember that when I entered this parish as a resident the nursing homes could be counted by units, and what is more, they were conducted in an irreproachable manner and were not a nuisance to private inhabitants, in consequence of the paucity of those institutions. What, however, does the old resident see now? He sees house after house intended by their construction as private residences transformed into so-called nursing homes with little or no provision for the sanitary disposal of hospital refuse. Moreover, many of these institutions are owned and directed by untrained women; that is to say, by women without any recognised medical training, and they are to be found employing as "nurses" their domestic servants, thus reflecting discredit on an honoured profession. In many instances the method utilised for the acquisition of such premises is to lease the house apparently as a private residence, but only a short time elapses before the in-coming tenant, with all possible secrecy, converts the premises into a so-called nursing home, and it is when the neighbouring private inhabitants notice the coming and going of patients, the sombrely-draped windows, the "nurses" now and then in uniform, and the heaps of malodorous straw placed in the street occasionally, that the unwelcome change—that is to say, unwelcome to the private residents—becomes generally known. To state that such institutions have appreciably contributed to the abuse of a nurse's uniform is to affirm what has long been obvious to many old residents in this parish. This is not all that can be said to the detriment of certain nursing homes in Marylebone, which by reason of their unhealthy influence have virtually expelled from the parish a large number of private residents. A much more serious charge that can be sustained against these questionable nursing institutions is that they have been known to admit infectious cases, and that they have received for treatment persons who, although not certifiably lunatics, have been so far demented as to be irresponsible for their actions. The statements in this last sentence are not exaggerations; they are strictly true, and to their accuracy many an old resident can testify. It is doubtless an advantage to have in proximity to Harley Street ample nursing facilities, but ought that to be a reason for the abuse of a nurse's uniform in certain institutions; for the multiplication of nursing homes to such an extent that life to the private resident is unhealthy and gloomy; and for the admission of cases that from their nature ought never to be found in such establishments? So great has been the increase of nursing homes in Marylebone that there is to be found one adjoining a baker's shop, and another over a grocer's. Is this desirable?

### Appointments.

[No charge is made for announcements under this head, and we are always glad to receive and publish appointments. The information, to insure accuracy, should be sent from the nurses themselves, and we cannot undertake to correct official announcements which may happen to be inaccurate. It is essential that in all cases the school of training should be given.]

Addenbrooke's Hospital, Cambridge.—Miss Alice Blomfield has been appointed matron. She was trained at the London Hospital, and was holiday sister in that institution. She has since been sister at Queen Charlotte's Hospital, London, and lady superintendent of the East End Mothers' Lying-in Home, Stepney.

Clevedon Cottage Hospital, Somerset.—Miss Effie Dobbell has been appointed matron, and Miss Melita Large, staff nurse. Miss Dobbell was trained at the Kent and Canterbury Hospital, and has since been staff nurse at Fulham Infirmary, charge nurse at Park Hospital, Lewisham; head nurse at Mylands Hospital, Colchester; matron of Cheriton Isolation Hospital, Folkestone; and deputy-matron of Hanwell Cottage Hospital. Miss Large was trained at Dorset County Hospital, and has since been staff nurse at Park Hospital, Lewisham; staff nurse at Mylands Hospital, Colchester; and charge nurse at Cheriton Isolation Hospital, Folkestone.

Eston Hospital, Yorkshire.—Miss Eveline Hannon has been appointed sister. She was trained at the Royal Hospital, Devonport, and has since been night charge nurse at Friedenheim Hospital, Swiss Cottage, London, and temporary sister in charge at the Isolation Hospital, Wimbledon.

Infectious Diseases Hospital, Strichen, Peterhead.—Miss Jessie R. Armstrong has been appointed matron. She was trained at the Town's Hospital, Glasgow, and has since been housekeeper at Ruchill Fever Hospital, Glasgow.

Paignton Cottage Hospital.—Miss Alice D. Brierley has been appointed matron. She was trained at Guy's Hospital, London, and the Greek Hospital, Alexandria, and has since been matron of the Tetbury Cottage Hospital, and the West Highland Cottage Hospital, Oban. She has recently been working in Malta for the Soldiers and Sailors' Families' Association.

Tunbridge Union Infirmary.—Miss Ethel Elizabeth Cave has been appointed charge nurse. She was trained Lewisham Union Infirmary, where she was afterwards char nurse.

### Presentations.

ROXBURGH DISTRICT ASYLUM, MELROSE.—Miss Janet Scott, who has been head nurse at Roxburgh District Asylum, Melrose, for nearly three years, was, on the occasion of her leaving that institution, presented with a writing-case and travelling-rug, as a mark of the respect and esteem in which she was held by the members of the staff.

### Novelties for Nurses.

(BY OUR SHOPPING CORRESPONDENT.)

### A CHEAP BAG FOR MATERNITY NURSES.

THIS bag is the outcome of an endeavour on the part of the Medical Supply Association, 228 Gray's Inn Road, to produce a fitted bag for maternity nurses and midwives at the lowest possible cost for the educational classes of the London County Council. The result is, that for 10s. 6d., nurses can now secure a strong and neat bag, with interchangeable lining, fitted with clinical thermometer, pulse-glass, syringe, nail-brush, scissors, bottles, ointment-jar, dredger, carbolic soap, mercury tablets, and thread. Naturally, for so small a cost, the fittings are of the simplest, but they are quite serviceable, and many will be glad to be spared a heavier outlay at the commencement of work, when considerable expenses have to be met and work is yet scarce. Supplementary fittings and appliances required in maternity nursing are supplied at very reasonable prices by the association.

### TRAVEL NOTES AND QUERIES.

BY OUR TRAVEL CORRESPONDENT.

NOTICE.—I shall be glad if those wanting help concerning their summer holidays will write to me in May, because I shall be on the continent in June and July, and not so well able to give satisfactory answers, besides the unavoidable delay consequent on the postal distance.

LYNTON AND LYNMOUTH (A. G. H.).—Many thanks for addresses, which will be most useful. For the case needed unfortunately much too expensive.

BELGIUM (Margaretta).—Thanks for your kind letter. I shall be glad to help further, but let me know as to time and money at your disposal, and something of your tastes.

BRITTANY FOR A MONTH (A. L.).—North Brittany is fairly bracing and most agreeable, but in the south it is a little enervating. Would you like the Bay of St. Malo? You could get accommodation there that would come within your means. Dinard itself is expensive, but I could tell you of accommodation in St. Servan, or St. Malo, or St. Enôgat, at from 6 to 7 francs per day. St. Servan is the most convenient centre for excursions You could from there visit Le Mont St. Michel and Dinan and make several smaller trips. Let me hear again when you want to go. June is cheaper than the later months.

PLOMBIÈRES, FRANCE (France).—Plombières is in the Department of Vosges. The season is from May to September. You must not think for a moment of taking your friend there or to any other health resort without the advice of your medical man. It may be described as a place beneficial to his complaint, and yet there may be reasons why in his particular case it would be unsuitable. Yes, there are good doctors there, but, in the event of your taking a patient for a cure there, the arrangement is for the English doctor to write a letter of introduction for the patient to take to one of the medical men of the place. A lack of French conversation would not cause any serious inconvenience; there are usually one or two waiters and chambermaids who speak a little English.

CHANNEL ISLANDS AND BRITTANY (C. B.).—The same objection in my mind applies to combining Brittany with the Channel Islands as would to joining Normandy to the Islands. It is attempting too much. If I remember rightly, you have only a fortnight at your disposal. The circular tour you speak of is certainly of interest, and Mont St. Michel is one of the gems of the Continent, but I should not attempt to do so much in so short a time. All your holiday will be spent rushing from place to place. Personally I prefer Brittany to the Channel Islands. Write me again fully as to time and money and whether you mean to combine Jersey and Guernsey and the circular tour, or rest content with the Islands alone or Brittany alone. I will then give you addresses.

NORTH CORNWALL (Hygiene).—I think Tintagel or Boscastle would suit. I believe there are golf links in both places, arranged in the last few years. A family whom I recommended to Tintagel under similar circumstances to yours were delighted with the Wharncliff Arms Hotel, a small old-fashioned hotel much frequented by artists and literary folk. There is a huge and hideous modern hotel, but very expensive, and without the home-like feeling of the one I recommend. I do not care for Port Isaac, nor for Perran Porth, but Boscastle, four miles north of Tintagel, is a charming little place, much more of a town than Tintagel. Hotel Wellington or Wivell's Private Hotel. If my suggestions do not suit I will think of something else.

A FORTNIGHT'S HOLIDAY IN PARIS (Clovelly).—The cheapest fare second class return to Paris is via Southampton and Havre, £2 0s. 8d.; via Newhaven and Dieppe, £2 2s. 3d. It is quite untrue that second class on continental railways is so wretched. I usually go third class myself, except when in a hurry, and I have frequently travelled in the fourth class in Germany and experienced no inconvenience. The idea that second class is impossible is simply an insular prejudice. Certainly you can manage on £5 per week, it is not necessary to spend so much. Hôtel St. Petersburg is rather dear and your bill would come to nearly 10s. per day, which is more than you should pay if you are to keep within £5 per week. The Hôtel Burgundy in the Rue Duphot, which is very central, would suit you better; or Hôtel Londres et de Milan in the Rue St. Hyacinthe. Ask for rooms on the third or fourth floor if you do not mind stairs. No, I do not like Cook's coupons, they are only useful for helpless people. You do not want pension terms, because you will usually be out in the middle of the day. Take early breakfast and late dinner in your hotel and get lunch at a Duval restaurant. A third hotel equally good would be the Pasquier, close to the Madeleine Your lack of French will be no trouble, almost all French hotels are accustomed to English clients. Tell me if I can help further.

HOLIDAY IN SWITZERLAND.—On the same lines as previously, I propose to organise another tour to Switzerland this summer. Previous tours having been so successful, I shall arrange to visit another part of the Bernese Oberland if a party is formed. We shall start on Tuesday, August 1st, and those coming straight back will arrive in London on Tuesday, August 15th, the holiday being organised for that fortnight. The cost will be 10 guineas. This includes second-class travelling from and back to London (third class on the Mountain Railway above Spiez) and full board (including breakfast, lunch, and dinner) and lodging at comfortable hotels. Expenses connected with luggage must be defrayed by the owners, and there may be a small charge for the drive between Frutigen and Kandersteg. The tickets will be available for 25 days for those wishing to extend their stay. The route outwards will be *via* Dover, Calais, Basle, Berne, and Spiez. We shall divide our time between Kandersteg and Zweisimmen, arriving there on Wednesday, August 2nd. On Tuesday, August 8th, the Kandersteg half of the party will go to Zweisimmen, and the Zweisimmen party to Kandersteg. The return journey will be commenced on Monday, August 14th, and will be *via* Montreux (where there will be time for a visit to Chillon Castle and a short walk along the Lake of Geneva) to Lausanne, thence to Paris, and back to London *via* Boulogne or Calais. Those who wish to prolong their stay can easily visit Geneva (and Chamounix) or can go up the Rhone Valley to Zermatt or other well-known centres. The journey can also be broken in Paris. Application should be made in May or June—as the party is limited in number, and the hotels fill early—to L. Edna Walter, B.Sc., Inspector to the Board of Education. 38, Woodberry Grove, Finsbury Park, London, N.

### Notes and Queries.

**REGULATIONS.**

**The Editor is always willing to answer in this column, without any fee, all reasonable questions, as soon as possible.**

**But the following rules must be carefully observed.**

1. **Every communication must be accompanied by the name and address of the writer.**
2. **The question must always bear upon nursing, directly or indirectly.**

**If an answer is required by letter a fee of half-a-crown must be enclosed with the note containing the inquiry.**

*Dispenser.*

(50) Can you tell me the best method of obtaining a position as dispenser, either in private practice or a public institution?—*N. R.*

You should advertise or answer advertisements, presuming, of course, that you are properly qualified.

*Massage.*

(51) Can you tell me of a seaside town where there would be an opening for a certified masseuse during the season? My doctor here would be pleased to recommend me to other medical men.—*Nurse B.*

We cannot tell you of any particular opening, but if you decide upon your locality and your doctor recommends you to other medical men it might be possible for you to become temporarily attached to some nursing institution or nursing home.

*Filling an Interval.*

(52) I should be glad to be advised as to the way a girl of 18, a lady, could most advantageously fill the three years before she is old enough to begin her training at a children's hospital. She has no home, and cannot spend much. After learning as much as possible of household management, cookery, etc., where would it be possible for her to be taken in return for her services?—*An Old Aunt.*

She might be taken in a Children's Convalescent Home or a consumptive sanatorium. "Burdett's Hospitals and Charities" gives a full list of such homes. It is obtainable from the Scientific Press, Ltd., 28 & 29 Southampton Street, Strand, W.C.

*Home or Institution.*

(53) Can you tell me of a home or institution where a poor young woman would be taken care of? She is strong and willing to work, but so forgetful, and at times so vacant.—*Anxious Sister.*

Write fully to the Secretary of the National Association for Promoting the Welfare of the Feeble-Minded, 53 Victoria Street, S.W.

*Bedsteads.*

(54) Can you give me any references to books or articles dealing with the advantages of iron or brass bedsteads over the old-fashioned wooden ones?—*F. W.*

It is simply a sanitary question.

*New York Diplomas.*

(55) Can you inform me if the diplomas received from hospitals in New York, after three years' training, are recognised in England?—*E.A.*

They would, of course, be "recognised" in any civilised part of the world. But it is often a little difficult for a nurse holding a New York certificate to start in England.

*Companion.*

(56) Can you recommmend a cheerful, companionable young lady to come here for a week? My sons and daughters are going away during that time. I am an old gentleman of 70, but require no nursing, although an invalid with heart trouble. . . . Let me know at once.—*R. H.*

We, of course, cannot recommend individuals. You can advertise, or answer advertisements, or go to a registry office for nurses, or a nurses' co-operation. We do not undertake to answer questions at once, unless a fee for that purpose is enclosed. See rules above.

**Handbooks for Nurses.**

| | Post Free. | |
|---|---|---|
| "How Become a Nurse: How and Where to Train." | 2s. | 4d. |
| "The Nurses' Dictionary" (Pronouncing) ... ... | 2s. | 0d. |
| "Nursing: its Theory and Practice" (Lewis.) ... ... | 3s. | 6d. |
| "The Light Treatment" (just published) ... ... | 2s. | 6d. |
| "A Complete Handbook of Midwifery." (Watson.) ... | 6s. | 4d. |

Of all booksellers or of the Scientific Press, Limited, 28 & 29 Southampton Street, Strand, London, W.C.

### For Reading to the Sick.

"BE WITH US, LORD."

THE Day is gently sinking to a close,
Fainter and yet more faint the sunlight glows;
O Brightness of Thy Father's Glory, Thou
Eternal Light of Light, be with us now;
Where Thou are present, Darkness cannot be,
Midnight is glorious Noon, O Lord, with Thee.

Our changeful lives are ebbing to an end,
Onward to darkness and to death we tend;
O Conqueror of the Grave, be Thou our Guide,
Be Thou our Light, in Death's dark Eventide;
Then in our mortal hour will be no gloom,
No sting in Death, no terror in the Tomb.

*Bishop Wordsworth.*

You will now have seen that it is on behalf of the weak among His people, that the power and grace of Christ are specially exerted. They are the tender among flocks, the bruised among reeds, the fearful among penitents, the feeble and erring among disciples, on whose behalf He delights to show Himself strong. His power rests upon infirmity; His strength is made perfect in weakness. Our spiritual life, to the last, may be one of feeble and ineffectual effort after a sanctity not reached; after a victory over the will, not perfected; after a grasp of faith not realised.

Again, this view of the tenderness of our Lord should be very comforting to us under the weakness of our faith, when we are laying hold of the promises of God, but not firmly; when, though professing to believe all the gracious assurances of the Holy One, the belief itself is not absolute and entire. Under such circumstances, what a consolation it is to know that the same weaknesses have been discovered by our brethren in the world, and that a gracious Saviour allowed for, excused, pardoned them! Faith in its lowest degree is precious; must be so, because, whatever its measure, it is the gift of God. And therefore to all who are suffering from this we say, "Be not afraid, only believe." If at the last day it should be said, that your heart was right, your eye was single, your desires were heavenward, your hope in the promise simple, scriptural, humble, pure, Christ will welcome you to a throne by the side of His most exalted saints, and, as He wipes away the last tear from your eyes, will say, "Why were ye fearful, O ye of little faith?"

*Rev. D. Moore.*

Enjoy the blessings of this day, if God sends them; and the evils of it bear patiently and sweetly: for this day is only ours, we are dead to yesterday, and we are not yet born to the morrow. But if we look abroad, and bring into one day's thoughts the evil of many, certain and uncertain, what will be and what will never be, our load will be as intolerable as it is unreasonable.—*Jeremy Taylor.*

As soon as we lay ourselves entirely at His feet, we have enough light given us to guide our own steps; as the foot-soldier, who hears nothing of the councils that determine the course of the great battle he is in, hears plainly enough the word of command which he must himself obey.

*George Eliot.*

